# Identification of potential pathways and microRNA-mRNA networks associated with benzene metabolite hydroquinone-induced hematotoxicity in human leukemia K562 cells

**DOI:** 10.1186/s40360-022-00556-8

**Published:** 2022-04-02

**Authors:** Chun-Hong Yu, Shui-Qing Yang, Lei Li, Yu Xin, Fang Zhang, Xiao-Fan Liu, Zong-Chun Yi

**Affiliations:** 1 School of Biological Science and Medical Engineering, Beihang University, Beijing, 100191 China; 2 School of Engineering Medicine, Beihang University, Beijing, 100191 China

**Keywords:** Hydroquinone, Transcriptomic, MiRNA, Hematotoxicity, K562 cells

## Abstract

**Background:**

Hydroquinone (HQ) is a phenolic metabolite of benzene with a potential risk for hematological disorders and hematotoxicity in humans. In the present study, an integrative analysis of microRNA (miRNA) and mRNA expressions was performed to identify potential pathways and miRNA-mRNA network associated with benzene metabolite hydroquinone-induced hematotoxicity.

**Methods:**

K562 cells were treated with 40 μM HQ for 72 h, mRNA and miRNA expression changes were examined using transcriptomic profiles and miRNA microarray, and then bioinformatics analysis was performed.

**Results:**

Out of all the differentially expressed genes (DEGs) and differentially expressed miRNAs (DEMs) induced by HQ, 1482 DEGs and 10 DEMs were up-regulated, and 1594 DEGs and 42 DEMs were down-regulated. HQ-induced DEGs were involved in oxidative stress, apoptosis, DNA methylation, histone acetylation and cellular response to leukemia inhibitory factor GO terms, as well as metabolic, Wnt/β-catenin, NF-κB, and leukemia-related pathways. The regulatory network of mRNAs and miRNAs includes 23 miRNAs, 1108 target genes, and 2304 potential miRNAs-mRNAs pairs. MiR-1246 and miR-224 had the potential to be major regulators in HQ-exposed K562 cells based on the miRNAs-mRNAs network.

**Conclusions:**

This study reinforces the use of in vitro model of HQ exposure and bioinformatic approaches to advance our knowledge on molecular mechanisms of benzene hematotoxicity at the RNA level.

**Supplementary Information:**

The online version contains supplementary material available at 10.1186/s40360-022-00556-8.

## Background

Hydroquinone (HQ) is a phenolic metabolite of benzene representing potential risks for hematological disorders and hematotoxicity [1–3]. Extensive research has shown that HQ may contribute to benzene-induced leukemia by oxidative stress, DNA damage, cell cycle regulation, and apoptosis [4–10]. HQ can inhibit erythroid differentiation in HD3 chicken erythroblast cells, K562 human leukemia cells, and U937 human leukemia cells [4, 11–14]. Our previous studies suggest that benzene metabolite phenol, 1,2,4-benzenetriol, and HQ considerably inhibit hemin-induced erythroid differentiation in K562 cells [14, 15]. The underlying molecular mechanisms involved in HQ toxicity are not fully understood.

MicroRNAs (miRNAs) are endogenous, highly conserved small non-coding RNAs that modulate various biological processes like cell survival, proliferation, metabolism, differentiation, and apoptosis [16–19]. The miRNA-mediated gene silencing can involve translational repression, co-translational protein degradation, competition for the cap structure, inhibition of ribosomal subunit joining, inhibition of mRNA circularization through deadenylation, or deadenylation-dependent decay [20, 21]. But recently, miRNAs have been found to mediate gene activation through the bidirectional transcription of the human genome, binding to the enhancer, or recruiting a protein complex with transcriptional activators to the gene promoter [22, 23]. Repression is more common in eukaryotes whereas posttranscriptional upregulation has been observed in specific cell types with distinct transcripts or conditions [23]. MiRNAs have been identified as exerting a powerful effect upon acute myeloid leukemia (AML) development and can be used as potential biomarkers for leukemia diagnosis and prognosis [24–26]. Various studies have assessed the role of miRNAs in particulate matter-exposed human pulmonary epithelial cells, sulfur mustard-resistance of the keratinocyte cell line, alternariol and altertoxin II-treated HepG2 cells, and environmental toxicants-exposed aquatic organisms to explore the corresponding toxicity mechanisms [27–30]. As Liang et al. point out, miRNA-451a and miRNA-486-5p expression are notably lower in HQ-treated CD34+ hematopoietic progenitor cells and K562 cells [13]. HQ may activate apoptotic signals via inhibiting the tumor-suppressive effects of miR-7-5p in TK6 lymphoblastoid cells [31]. However, the role of miRNAs and their target mRNAs expression in benzene hematotoxicity has not been fully addressed yet and needs further study.

Transcriptome sequencing with high sensitivity and good reproducibility is a bargain for detecting low-expression genes [32]. The integration of genomic tools contributes to investigating the molecular mechanism of toxicity [33]. Human leukemia K562 cells were derived from a patient with chronic myeloid leukemia [34]. Here we performed transcriptomic profiles and miRNA microarray to identify mRNAs and miRNAs changes, constructed the mRNAs and miRNAs regulatory network, and performed an in-depth bioinformatic analysis in HQ-induced K562 cells. The differentially expressed mRNAs and miRNAs participated in the metabolic pathways, Wnt/β-catenin pathway, NF-κB pathway, etc. by regulating oxidative stress, apoptosis, DNA methylation, histone acetylation, resulting in hematotoxicity including leukemia. These findings provided a theoretical basis for understanding the molecular mechanisms of benzene hematotoxicity, laying the foundation for future validation of in vivo models as well as therapeutic targets and prognostic factors.

## Methods

### Cell culture

Human leukemia K562 cells were purchased from Cell Resource Center, PekingUnion Medical College (CRC/PUMC, China), and were cultured as described previously [15, 34]. After K562 cells were treated with 40 μM HQ (Sigma-Aldrich) for 72 h, the cells were harvested for further study.

### Transcriptome analysis

The genome-wide transcriptome analysis was analyzed as described previously [35]. Differentially expressed genes (DEGs) analysis was indicated with the absolute log_2_ (fold change of HQ/C) values and the adjusted *P*-value <0.05 was considered differentially expressed. Data are representative of three independent experiments. The volcano plot of DEGs was performed by creating scatter plots in Excel software. Select the data of downregulated DEGs including log_2_ (fold change of HQ/C) values and the adjusted *P*-value; choose the scatter plot to represent the relationship between the data sets; Add green color for the dots. The data of upregulated DEGs and genes without differential changes were performed in the same plot and added red and blue colors, respectively. Gene Ontology (GO) enrichment analysis of DEGs was conducted by over-representation analysis (ORA) using the online database WebGestalt (http://www.webgestalt.org). Significantly enriched GO terms in DEGs compared to the genome background were defined by Wallenius’ non-central hypergeometric distribution adjusting for gene length bias [36]. The pathway analysis of DEGs was conducted using the Kyoto Encyclopedia of Genes and Genomes (KEGG) database (http://www.kegg.jp/) [37].

### miRNA microarray analysis

The miRNA microarray was analyzed as described previously [38]. Total RNA was extracted with Ribozol™ RNA Extraction Reagent (Invitrogen, USA) according to the manufacturer’s protocol. The expression of microRNAs contained in the miRBase was analyzed by microarray using the μParaflo™ Microfluidic Biochip Technology of LC sciences (Huston, TX, USA). A total of 1 μg of miRNA-enriched RNA was labeled with Cy3 using the ULS™ microRNA labeling kit (Kreatech, USA) and hybridized on the microarray. Hybridization images were collected using a laser scanner and digitized using Array-Pro image analysis software. The signals were normalized using a locally-weighted regression (LOWESS) filter.

Differentially expressed miRNAs (DEMs) analysis was indicated with the absolute log_2_ (fold change of HQ/C) values and *P*-value <0.05 was considered differentially expressed. Data are representative of three independent experiments. Hierarchical clustering analysis of the DEMs was performed and graphs were generated using the ggplot2 package in R software.

### miRNA-mRNA regulatory network construction

The potential target mRNAs of miRNAs were screened from miRWalk (http://mirwalk.umm.uni-heidelberg.de), miRanda (https://microbit.org), TargetScan (http://www.targetscan.org), miRDB (http://mirdb.org) and RNA22 (https://cm.jefferson.edu). The miRNA target genes that appeared in at least three prediction databases were screened and quantified from the results of transcriptome analysis. Cytoscape software (version 3.8.0) was employed to construct and analyze the miRNA-mRNA regulatory network.

## Results

### The effects of HQ on DEGs and DEMs in K562 cells

**Fig. 1:**
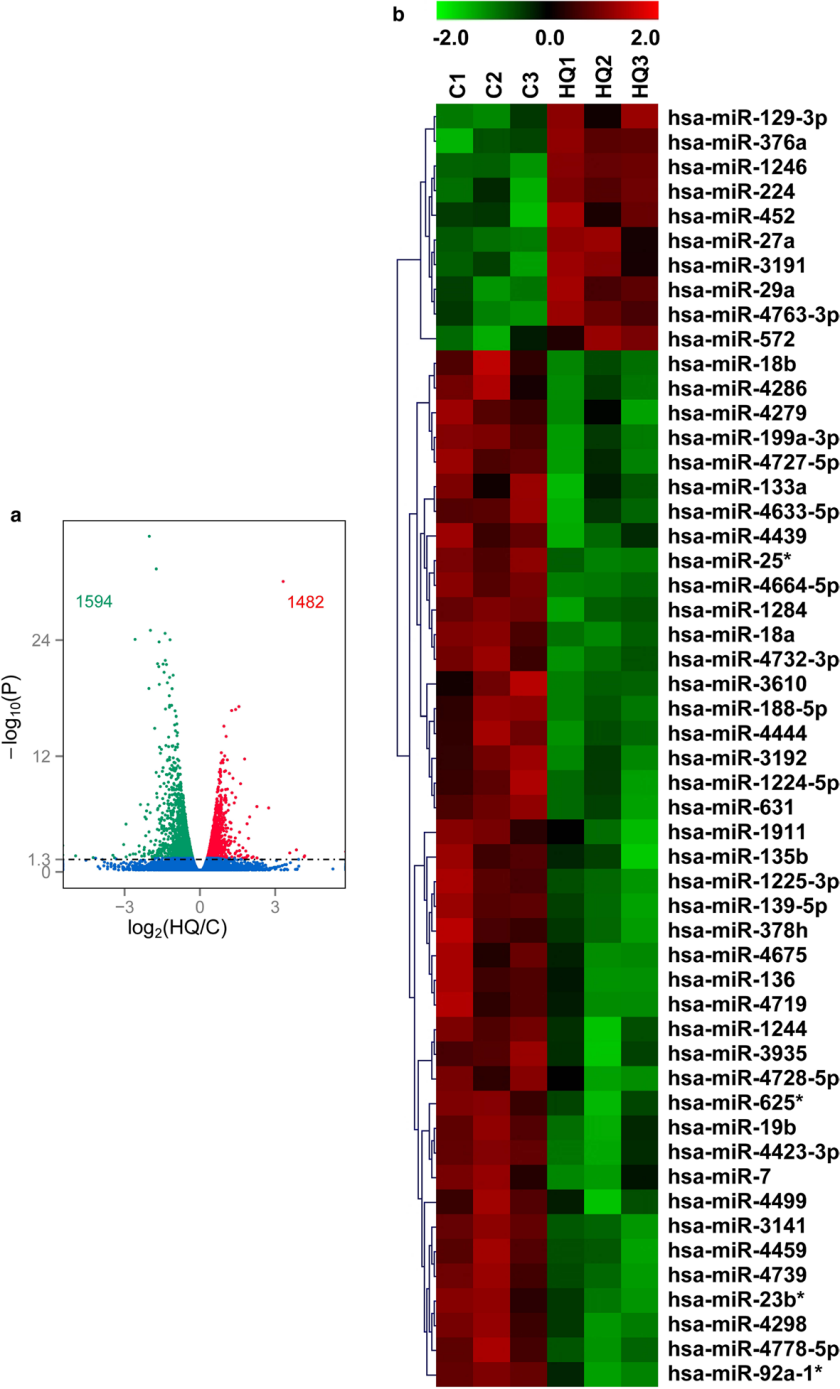
DEGs and DEMs in HQ-induced K562 cells. **a** The volcano plot displays the gene expression in the HQ group compared with control by transcriptomic analysis. Each point represents one of the detected genes. Green points are downregulated DEGs, red points are upregulated DEGs, and blue points are genes without differential changes when compared with control. Horizontal dotted lines indicate statistical thresholds for adjusted *P*-value <0.05. **b** The clustered heatmap of HQ and C groups by miRNA microarray assay. HQ: hydroquinone-induced K562 cells; C: the control group

The volcano plot of overall gene expression showed that 3076 genes were differentially expressed in K562 cells treated with 40 μM HQ for 72 h, including 1482 upregulated and 1594 downregulated DEGs (Fig. 1a). Moreover, there were 84 upregulated and 307 downregulated DEGs over a 2-fold change after HQ exposure. The top 10 upregulated DEGs and top 10 downregulated DEGs based on the absolute log_2_ (fold change of HQ/C) values after HQ exposure for 72 h were set out in Table 1. What stands out in the table is that *LILRA6* (log_2_ (HQ/C) = 4.17) was the most upregulated DEG and *HBB* (log_2_ (HQ/C) = −4.95) was the most downregulated DEG.

**Table 1:** Top 10 upregulated or downregulated DEGs in HQ-induced K562 cells

Gene name	Description	C	HQ	Log_2_ (HQ/C)	Adjusted *P*-value
*LILRA6*	leukocyte immunoglobulin like receptor A6	0.4	6.4	4.17	0.025*
*KISS1*	KiSS-1 metastasis-suppressor	0.4	6.2	4.15	0.031*
*GPR35*	G protein-coupled receptor 35	0.7	10.1	3.84	0.006**
*CREB3L3*	cAMP responsive element binding protein 3 like 3	0.7	8.5	3.58	0.012*
*THBS1*	thrombospondin 1	31.8	317.2	3.32	0.000***
*PI16*	peptidase inhibitor 16	6.7	44.9	2.74	0.000***
*CCL2*	C-C motif chemokine ligand 2	11.7	56.3	2.27	0.000***
*IGFBP5*	insulin like growth factor binding protein 5	18.4	80.3	2.13	0.031*
*CHGB*	chromogranin B	2.8	11.9	2.07	0.037*
*ITGA3*	integrin subunit alpha 3	11.3	44.7	1.98	0.000***
*KIF19*	kinesin family member 19	10.6	1.8	−2.57	0.018*
*BMP2*	bone morphogenetic protein 2	9.9	1.6	−2.66	0.018*
*LRRTM1*	leucine rich repeat transmembrane neuronal 1	8.9	1.3	−2.79	0.023*
*NMUR1*	neuromedin U receptor 1	21.9	2.9	−2.94	0.000***
*MARVELD3*	MARVEL domain containing 3	7.4	1.0	−2.94	0.037*
*ACTL10*	actin like 10	12.7	1.5	−3.04	0.002***
*LRRC14B*	leucine rich repeat containing 14B	6.4	0.6	−3.41	0.046*
*KRT36*	keratin 36	7.1	0.6	−3.47	0.024*
*GOLGA8Q*	golgin subfamily A	5.7	0.3	−4.25	0.045*
*HBB*	hemoglobin subunit beta	370.6	12.0	−4.95	0.029*

* represents *P* value < 0.05; ** represents *P* value < 0.01; *** represents *P* value < 0.005

The heatmap and hierarchical clustering analysis showed that 52 miRNAs were differentially expressed after HQ exposure, including 10 upregulated and 42 downregulated DEMs (Fig. 1b). In addition, 7 upregulated and 23 downregulated DEMs were over 2-fold change after HQ exposure. These results indicated that majority of genes and miRNAs were downregulated after HQ exposure. The top 10 upregulated DEMs and top 10 downregulated DEMs based on the absolute log_2_ (fold change of HQ/C) values after HQ exposure for 72 h were set out in Table 2. It is apparent from this table that miR-1246 (log_2_ (HQ/C) = 4.49) was the most upregulated DEM and miR-4423-3p was the most downregulated DEM.

**Table 2:** Top 10 upregulated or downregulated DEMs in HQ-induced K562 cells

miRNA name	C	HQ	Log_2_ (HQ/C)	*P*-value
miR-1246	1278	28,646	4.49	0.001***
miR-224	251	909	1.86	0.034*
miR-572	51	150	1.55	0.033*
miR-129-3p	63	166	1.41	0.026*
miR-29a	86	219	1.35	0.011*
miR-27a	128	291	1.18	0.039*
miR-452	22	49	1.16	0.045*
miR-376a	24	40	0.77	0.030*
miR-3191	25	41	0.75	0.025*
miR-4763-3p	70	90	0.36	0.009**
miR-4778-5p	4845	1973	−1.3	0.008**
miR-4298	4588	1859	−1.3	0.011*
miR-23b*	28	12	−1.3	0.015*
miR-4732-3p	34	14	−1.32	0.007**
miR-7	158	58	−1.46	0.032*
miR-135b	28	10	−1.48	0.043*
miR-18a	1917	661	−1.54	0.002***
miR-92a-1*	118	38	−1.65	0.030*
miR-25*	209	66	−1.66	0.002***
miR-4423-3p	81	14	−2.58	0.028*

* represents *P* value < 0.05; ** represents *P* value < 0.01; *** represents *P* value < 0.005

### DEGs involved in the oxidative stress, apoptosis, DNA methylation, histone acetylation and cellular response to leukemia inhibitory factor GO terms in HQ-induced K562 cells

**Fig. 2:**
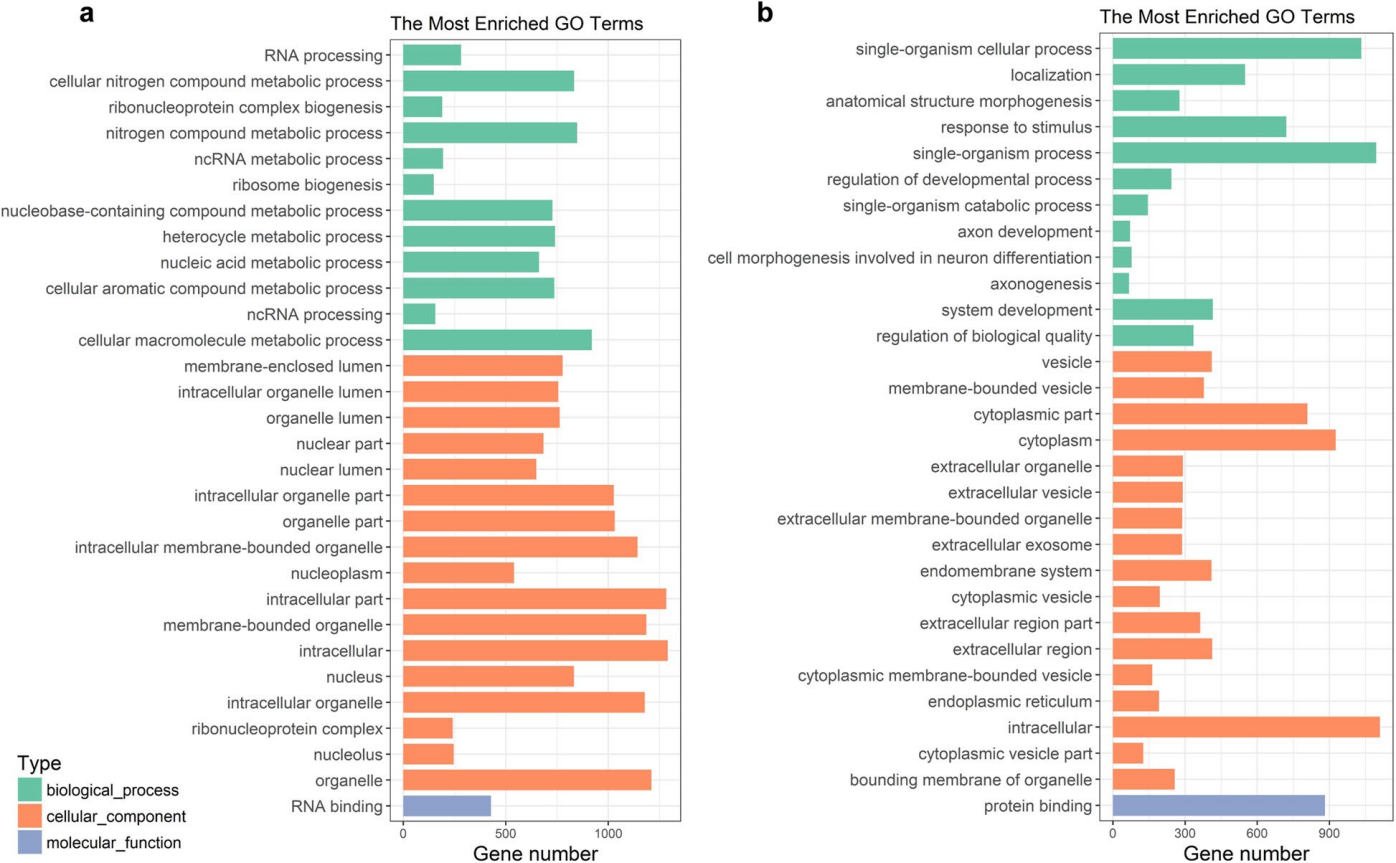
GO analysis of DEGs in HQ-induced K562 cells. GO enrichment histogram of upregulated DEGs (**a**) and downregulated DEGs (**b**)

The top 30 enriched GO terms of target DEGs were presented in Fig. 2. GO analysis showed that HQ-upregulated DEGs enriched in biological processes for RNA processing, cellular nitrogen compound metabolic process, and ribonucleoprotein complex biogenesis, whereas HQ-downregulated DEGs enriched in biological processes for single-organism cellular process, localization, and response to stimulus. Our previous studies have demonstrated that DNA methylation, histone acetylation, and ROS have major influences on the transcription of erythroid-specific genes [15, 39–41]. Here, we focused on the GO terms of oxidative stress, apoptosis, DNA methylation, and histone acetylation in HQ-induced K562 cells.

HQ treatment considerably increases the relative ROS levels in HD3 chicken erythroblast cells, HL-60 promyelocytic leukemia cells (HL-60 cells), Jurkat T-lymphoblastic leukemia cells (Jurkat cells), and K562 cells [4, 40, 42, 43]. In the present study, there were 38 upregulated DEGs and 37 downregulated DEGs in the response to oxidative stress term (GO:0006979), among which there were 27 upregulated DEGs and 20 downregulated DEGs in the cellular response to oxidative stress term (GO:0034599) in HQ-induced K562 cells. Taken together, these indicated the crucial role of oxidative stress in HQ exposure in K562 cells.

Activation of *CASP3* and *CASP8* both have pivotal roles in the execution phase of cell apoptosis. As mentioned, HQ induces apoptosis in HL-60 cells, Jurkat cells, human bone marrow mononuclear cells (BMMNC), and K562 cells accompanied by *CASP3* or *CASP8* activation [7, 44, 45]. Consistently, HQ exposure markedly upregulated the mRNA levels of *CASP3* and *CASP8* to 1.53-fold and 1.38-fold of that in the control K562 cells in the present study. Moreover, there were 17 upregulated DEGs and 14 downregulated DEGs in the positive regulation of apoptotic signaling pathway (GO:2001235), contributing to HQ-induced apoptosis in K562 cells.

Exposure to benzene metabolites phenol and HQ cause an increase in DNA methylation levels at some erythroid-specific genes clusters, including *α-globin*, *β-globin*, and *GATA1* gene clusters in K562 cells [39, 46]. There were 5 upregulated DEGs (*GATAD2A*, *PRMT5*, *BRCA1*, *BEND3*, and *MTRR*) and 2 downregulated DEGs (*TDRKH* and *SPI1*) in the DNA methylation term (GO:0006306) in HQ-induced K562 cells in the present study. To date, we have viewed no data on the effects of HQ on histone acetylation, but the effects of environmental chemicals on histone modifications have been explored recently [47, 48]. Presently, there were 17 upregulated DEGs (*LIF*, *BEND3*, *ATG5*, *NAA50*, *HAT1*, *TAF5L*, *POLE3*, *POLE4*, *GTF3C4*, *NOC2L*, *ING3*, *BRCA1*, *KANSL2*, *WDR5*, *RUVBL1*, *ACTL6A*, *SET*, and *BEND3*) and 5 downregulated DEGs (*SPI1*, *MAPK3*, *WBP2*, *FLCN*, and *YEATS2*) in the histone acetylation term (GO:0016573) in HQ-induced K562 cells, indicating their potential roles in HQ exposure.

Exposure to HQ can markedly change the mRNA levels and DNA methylation levels of erythroid-specific genes, as well as reactive oxygen species (ROS) levels in K562 cells [14, 39–41]. Transcriptomic analysis contributed to high-throughput data for HQ-induced DEGs in K562 cells, which further support the role of oxidative stress, apoptosis, DNA methylation, histone acetylation in benzene metabolite HQ-induced hematotoxicity. In addition, there were 20 upregulated DEGs and 10 downregulated DEGs in the cellular response to leukemia inhibitory factor term (GO:1990830) in HQ-induced K562 cells (Table 3). Different biological processes in response to HQ exposure might result in these genes expression changes and take part in the mechanism of benzene-induced leukemia.

**Table 3:** DEGs in the GO term of cellular response to leukemia inhibitory factor

Gene name	Description	C	HQ	Log_2_ (HQ/C)	Adjusted *P*-value
*INA*	internexin neuronal intermediate filament protein alpha	8	25	1.71	0.003***
*NEFH*	neurofilament heavy polypeptide	201	429	1.10	0.000***
*BCLAF1*	BCL2 associated transcription factor 1	868	1692	0.96	0.000***
*TWISTNB*	TWIST neighbor	232	448	0.95	0.009**
*NUP35*	nucleoporin 35	119	217	0.87	0.001***
*ARID5B*	AT-rich interaction domain 5B	51	93	0.87	0.002***
*WDR35*	WD repeat domain 35	138	242	0.80	0.000***
*MRPL15*	mitochondrial ribosomal protein L15	750	1194	0.67	0.000***
*NCL*	nucleolin	10,212	16,137	0.66	0.000***
*MAT2A*	methionine adenosyltransferase 2A	1673	2554	0.61	0.000***
*SRM*	spermidine synthase	3782	5646	0.58	0.003***
*RIF1*	replication timing regulatory factor 1	597	890	0.57	0.007**
*SRSF3*	serine and arginine rich splicing factor 3	4876	7227	0.57	0.000***
*CACYBP*	calcyclin binding protein	3052	4434	0.54	0.000***
*MRAS*	muscle RAS oncogene homolog	103	150	0.53	0.031*
*HNRNPU*	heterogeneous nuclear ribonucleoprotein U	8697	12,532	0.53	0.000***
*EED*	embryonic ectoderm development	412	585	0.51	0.003***
*SRSF7*	serine and arginine rich splicing factor 7	2457	3430	0.48	0.000***
*PDCD10*	programmed cell death 10	609	809	0.41	0.009**
*B3GNT2*	beta-1,3-N-acetylglucosaminyltransferase 2	468	598	0.35	0.047*
*LAPTM5*	lysosomal protein transmembrane 5	1844	1483	−0.31	0.040*
*KDM3A*	lysine demethylase 3A	895	703	−0.35	0.040*
*PFKP*	phosphofructokinase, platelet	3315	2523	−0.39	0.038*
*FZD4*	frizzled class receptor 4	331	218	−0.60	0.008**
*PARP16*	poly (ADP-ribose) polymerase family member 16	496	324	−0.62	0.000***
*PCOLCE*	procollagen C-endopeptidase enhancer	580	361	−0.68	0.000***
*SYNGR1*	synaptogyrin 1	693	360	−0.94	0.000***
*NR5A2*	nuclear receptor subfamily 5 group A member 2	40	20	−1.02	0.024*
*CTH*	cystathionine gamma-lyase	410	198	−1.05	0.000***
*BSPRY*	B-box and SPRY domain containing	53	22	−1.27	0.001***

* represents *P* value < 0.05; ** represents *P* value < 0.01; *** represents *P* value < 0.005

### DEGs involved in the metabolic, Wnt/β-catenin, NF-κB, and leukemia-related pathways in HQ-induced K562 cells

**Fig. 3:**
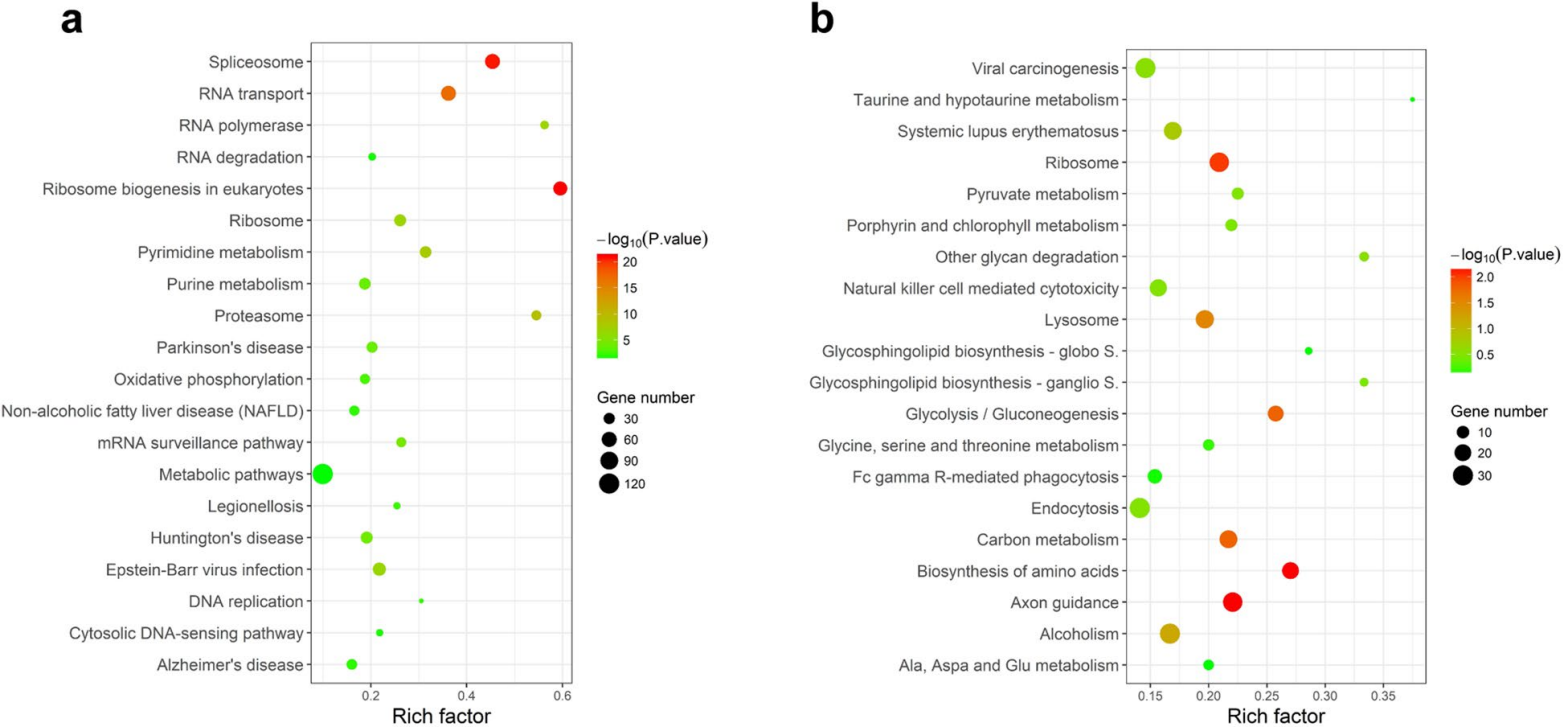
KEGG analysis for DEGs in HQ-induced K562 cells. KEGG pathway enrichment analysis of upregulated DEGs (**a**) and downregulated DEGs (**b**). Rich factor indicates the ratio of annotated DEGs number and whole background genes number in the corresponding pathway. The size of each bubble corresponds to the number of annotated DEGs, and the color gradient depicts the adjusted *P*-value of enrichment significance

The HQ-upregulated DEGs were annotated with 243 KEGG pathway whereas the downregulated DEGs included 262 KEGG pathway. The top 20 enriched pathways were presented in Fig. 3. KEGG pathway analysis demonstrated that HQ-upregulated DEGs were the most significantly enriched in the ribosome biogenesis in the eukaryotes pathway (annotated 50 DEGs, *P* = 4.03E-24), spliceosome, RNA transport, and proteasome whereas HQ-downregulated DEGs enriched in the axon guidance pathway (annotated 28 DEGs, *P* = 4.96E-05), biosynthesis of amino acids, ribosome and carbon metabolism (Fig. 3).

In addition, most genes were annotated as the metabolic pathways, attaining 121genes in both the upregulated and the downregulated DEGs. The cytochrome P450 family 1 subfamily A member 1 (*CYP1A1*) participates in metabolic pathways. The frizzled class receptor 2 (*FZD2*) gene encodes a protein in the beta-catenin canonical signaling pathway. HQ has been reported to considerably upregulate the mRNA level of *CYP1A1*, whereas downregulate the mRNA level of *FZD2* in human epidermal keratinocytes cells (HEK cells) [49]. In our results, the mRNA levels of *CYP1A1* and *FZD2* in K562 cells showed similar trends to the HEK cells.

The canonical Wnt/β-catenin pathway is a key factor in multiple biological processes like cell proliferation, apoptosis, motility, and differentiation. HQ treatment reduces the level of β-catenin, a dominant part in the canonical Wnt signaling pathway in BMMNC cells [45]. Presently, HQ considerably downregulated the mRNA levels of *WNT6* and *WNT11*, as well as other 12 genes (*FZD2*, *PLCB2*, *VANGL2*, *NFATC4*, *FZD4*, *FRAT1*, *PPARD*, *CTNNBIP1*, *RAC2*, *LRP5*, *FRAT2*, and *NFATC2*) in the Wnt signaling pathway (hsa04310) (Supplementary Fig. 1).

RNA-seq has identified that hydroquinone promotes DNA homologous recombination repair via activating the NF-κB pathway in the human osteosarcoma cell line (U2OS/DR-GFP) [50]. In our study, the mRNA level of *NFKB1* was upregulated after HQ exposure, indicating the NF-κB pathway might be activated in HQ-induced K562 cells.

Furthermore, there were 10, 12, 13, and 39 DEGs annotated as the KEGG terms of chronic myeloid leukemia, acute myeloid leukemia, hematopoietic cell lineage, and human T-cell leukemia virus 1 infection in HQ-induced K562 cells, respectively (Table 4) [37]. The related DEGs were highlighted in the KEGG maps in Supplementary Fig. 2–5. HQ exposure might change these genes’ expression to activate the leukemia-related pathways, resulting in potential benzene hematotoxicity.

**Table 4:** KEGG analysis of leukemia related pathways

KEGG term	ID	Input number	Background number	Regulated	Genes
Chronic myeloid leukemia	hsa05220	4	73	up	*CRK, CHUK, NFKB1, NRAS*
		6	73	down	*STAT5B, MAP 2 K2, PIK3CD, GAB2, SHC2, MAPK3*
Acute myeloid leukemia	hsa05221	4	57	up	*KIT, CHUK, NFKB1, NRAS*
		8	57	down	*PPARD, MAP2K2, SPI1, PIM1, PIK3CD, STAT5B, MAPK3, STAT3*
Hematopoietic cell lineage	hsa04640	4	88	up	*KIT, CD44, THPO, ITGA3*
		9	88	down	*ITGAM, ITGA2B, CD19, ANPEP, CD33, CD7, IL9R, CD37, ITGA5*
Human T-cell leukemia virus 1 infection	hsa05166	17	261	up	*PCNA, ANAPC10, CDC23, NFKB1, POLE3, FOSL1, ATR, VAC14, CDC26, CHUK, ETS2, POLE4, RANBP1, VDAC3, MRAS, CREM, NRAS*
		22	261	down	*STAT5B, HLA-E, TP53INP1, HLA-A, NFATC2, TNFRSF13C, FZD4, FZD2, IL15RA, PIK3CD, TLN1, NFATC4, HLA-B, WNT11, ATF3, SPI1, RRAS, MAP3K3, WNT6, HLA-C, TSPO, SLC25A6*

### Identification of miR-1246 and miR-224 as major regulators in the miRNA-mRNA network in HQ-induced K562 cells

**Fig. 4:**
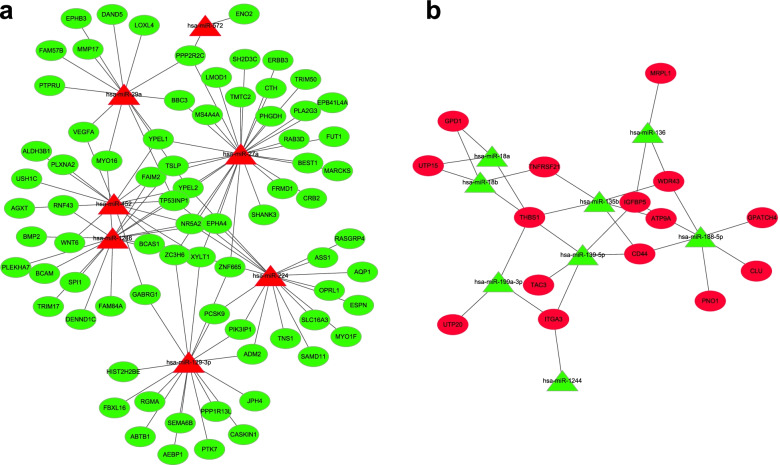
The miRNA-mRNA network of HQ-induced K562 cells. **a** The network of upregulated DEMs and downregulated target DEGs. **b** The network of downregulated DEMs and upregulated target DEGs. Triangles indicate DEM and circles indicate DEG. Red represents upregulated expression and green represents downregulated expression

The mutual regulation of DEMs and DEGs was analyzed to construct an interaction network after HQ exposure in K562 cells. It is a widely held view that miRNAs can induce the degradation of their target mRNAs, so we focused on those miRNAs and mRNAs with opposite expression trends. There were 10 upregulated DEMs and 1594 downregulated DEGs, as well as 42 downregulated DEMs and 1482 upregulated DEGs in HQ-induced K562 cells. The entire analysis led to the identification of 23 miRNAs, 1108 target genes, and 2304 potential miRNAs-mRNAs pairs in the network. Moreover, the miRNA-mRNA network of DEGs and DEMs over a 2-fold change was constructed by a cystoscope (Fig. 4).

The miRNA-mRNA network was constructed with 109 potential pairs containing 7 upregulated DEMs and 75 downregulated target DEGs in Fig. 4a; the network had 30 potential pairs containing 8 upregulated DEMs and 15 downregulated target DEGs in Fig. 4b. Per miRNA had 2–29 target genes and per gene was connected to 1–5 miRNAs. The lists of top miRNAs and target genes in the network were shown in Table 5 and Table 6. On the other hand, we found that majority of DEGs and DEMs were downregulated after HQ exposure.

**Table 5:** List of top-regulated miRNAs in the miRNA-mRNA regulatory network

miRNA name	Degree	Regulated
miR-27a	29	up
miR-1246	17	up
miR-129-3p	17	up
miR-224	17	up
miR-452	14	up
miR-29a	13	up
miR-572	2	up
miR-188-5p	6	down
miR-139-5p	5	down
miR-135b	5	down
miR-18b	4	down
miR-136	3	down
miR-199a-3p	3	down
miR-18a	3	down

**Table 6:** List of top-regulated genes in the miRNA-mRNA regulatory network

Gene name	Description	Degree	Regulated
*YPEL2*	protein yippee-like 2	5	down
*TP53INP1*	tumor protein p53-inducible nuclear protein 1	5	down
*XYLT1*	xylosyltransferase 1	4	down
*ZC3H6*	zinc finger CCCH domain-containing protein 6	4	down
*NR5A2*	nuclear receptor subfamily 5 group A member 2	4	down
*PPP2R2C*	serine/threonine-protein phosphatase 2A subunit B gamma isoform	3	down
*FAIM2*	Fas apoptotic inhibitory molecule 2	3	down
*EPHA4*	ephrin type-A receptor 4	3	down
*YPEL1*	protein yippee-like 1	3	down
*THBS1*	thrombospondin-1	5	up
*CD44*	CD44 antigen	3	up
*IGFBP5*	insulin like growth factor binding protein 5	3	up
*ITGA3*	integrin subunit alpha 3	3	up
*WDR43*	WD repeat-containing protein 43	3	up

HMDD (the Human microRNA Disease Database) is a database for experiment-based miRNA and human disease associations (http://www.cuilab.cn/hmdd) [51]. When the DEMs over a 2-fold change were screened from the HMDD 3.2, 5 miRNAs (miR-27a, miR-224, miR-1246, miR-18a, and miR-18b) were associated with leukemia, indicating potential roles in benzene-induced leukemia. According to the miRNA-mRNA regulatory network, there were more interactions between upregulated miRNAs and downregulated genes than the other way around. In our study, miR-1246 and miR-224 were the most upregulated DEMs with 17 target DEGs whereas miR-18a and miR-18b were downregulated in HQ-induced K562 cells. However, it remains unknown why MiR-27a was upregulated in HQ-induced K562 cells but downregulated in acute leukemia cell lines and primary samples compared to hematopoietic stem-progenitor cells [52]. Therefore, miR-1246 and miR-224 were identified to be major regulators for HQ exposure in K562 cells. These results indicated the crucial role of miR-1246 and miR-224 in benzene hematotoxicity.

## Discussion

Our previous studies have demonstrated that hemin-induced erythroid differentiation is concentration-dependently and time-dependently inhibited by benzene metabolites exposure (phenol, 1,2,4-benzenetriol, and hydroquinone) in K562 cells [14, 15, 39, 46]. In the present study, the concentration of 40 μM HQ was selected to correspond to no obvious cytotoxicity but markedly inhibiting erythroid differentiation in K562 cells when exposed for 72 h [14]. Hemoglobin subunit beta (*HBB*) loci are associated with beta-thalassemia, sickle cell anemia, and heinz body anemias [53–55] . *HBB* was the most dramatically downregulated gene after HQ exposure, which might play important roles in benzene hematotoxicity.

It has been reported that the NF-κB pathway is activated in the development of chronic myeloid leukemia and acute myeloid leukemia [56–59]. In our study, HQ exposure upregulated the mRNA level of *NFKB1* in chronic myeloid leukemia and acute myeloid leukemia pathway, which partly supported that HQ might promote leukemia development by activating the NF-κB pathway. HQ exposure downregulated the mRNA levels of *MAP 2 K2* and *MAPK3* in chronic myeloid leukemia and acute myeloid leukemia pathway (Table 4). This may inhibit the MAPK signaling pathway and thus inhibit cell proliferation (Supplementary Fig. 2, 3). These results were consistent with our previous study that HQ induced a concentration-dependent decrease in the viabilities in K562 cells [44]. The roles of metabolic pathways, Wnt/β-catenin pathway, and NF-κB pathway in benzene hematotoxicity need further study.

The downregulated miR-1246/1248 are key nodes that reveal the possible relapse mechanisms for pediatric T cell acute lymphoblastic leukemia. As reported, miR-1246 is one of the most highly enriched miRNAs in AML derived extracellular vesicles [16, 60, 61]. MiR-1246, a hundreds-fold alteration in microvesicles from three different leukemia cell lines (K562, Nalm-6, and Jurkat), is upregulated to activate the expression of C6orf211 and C19orf10 to promote tumor progression in patients diagnosed as chronic myeloid leukemia [62]. MiR-224 expression is considerably upregulated in the bone marrow of pediatric AML patients and can be used as noninvasive biomarkers for the early prediction of hepatocellular carcinoma development [63]. These results indicated the crucial role of miR-1246 and miR-224 in hematotoxicity. MiR-29a is highly expressed in arsenic-induced peripheral neuropathy, which is consistent with higher expression after HQ exposure in this study [64]. Low expression of miR-18a distinguishes pediatric and adult acute lymphoblastic leukemia from each other [65]. PML/RARα-regulated miR-181a/b cluster targets the tumor suppressor RASSF1A in acute promyelocytic leukemia [66].

In further research, the expression, target genes, and biological function of miR-1246 and miR-224 will be confirmed by more techniques in HQ-induced K562 cells, CD34+ hematopoietic progenitor cells, U937 human leukemia cells, and human peripheral blood mononuclear cells (PBMCs). Inhibition of miR-1246 and miR-224 will contribute to their regulation for HQ exposure. On the other hand, hydroquinone has been reported to inhibit PRV infection in mouse neuroblastoma N2a cells, protect neurons from transient cerebral ischemia, and reduce gliosis in a gerbil model of transient cerebral ischemia [67, 68]. The study on the effect of HQ on neural systems will contribute to a full understanding of benzene toxicity. Further assessment in suitable animal models or any data of miRNAs expression of benzene-exposed patients will be immensely beneficial to further studies.

## Conclusion

In summary, the present study identified differentially expressed genes and miRNAs in HQ-induced K562 cells using transcriptomic profiles and miRNA microarray. The miRNA-mRNA network can help us better understand the molecular mechanisms between miRNAs and their target genes. MiR-1246 and miR-224 had the potential to be major regulators for HQ exposure in K562 cells based on the miRNAs-mRNAs network and were reported to be associated with leukemia, suggesting potential biomarkers for the evaluation of benzene hematotoxicity. Apart from the miRNAs-mRNAs regulation, how miRNAs regulate protein expression remains elusive. Further proteomics study is to be performed to elucidate the underlying mechanism. The GO and KEGG pathways provide a framework for further studies in suitable in vitro and animal models, which will contribute to developing new strategies for the prevention and rapid diagnosis of benzene hematotoxicity.

## Supplementary Information

**Additional file 1: Supplementary Figure 1**. HQ-regulated DEGs of K562 cells in the Wnt signaling pathway. The Wnt signaling pathway was downloaded from the KEGG database (https://www.kegg.jp/kegg-bin/show_pathway?map04310). In K562 cells, HQ-upregulated DEGs were colored pink and HQ-downregulated DEGs were colored green. **Supplementary Figure 2**. HQ-regulated DEGs of K562 cells in chronic myeloid leukemia pathway. The chronic myeloid leukemia pathway was downloaded from the KEGG database (https://www.kegg.jp/kegg-bin/show_pathway?map05220). In K562 cells, HQ-upregulated DEGs were colored pink and HQ-downregulated DEGs were colored green. **Supplementary Figure 3**. HQ-regulated DEGs of K562 cells in acute myeloid leukemia pathway. The acute myeloid leukemia pathway was downloaded from the KEGG database (https://www.kegg.jp/kegg-bin/show_pathway?map05221). In K562 cells, HQ-upregulated DEGs were colored pink and HQ-downregulated DEGs were colored green. **Supplementary Figure 4**. HQ-regulated DEGs of K562 cells in hematopoietic cell lineage pathway. The hematopoietic cell lineage pathway was downloaded from the KEGG database (https://www.kegg.jp/kegg-bin/show_pathway?map04640). In K562 cells, HQ-upregulated DEGs were colored pink and HQ-downregulated DEGs were colored green. **Supplementary Figure 5**. HQ-regulated DEGs of K562 cells in human T-cell leukemia virus 1 infection pathway. The human T-cell leukemia virus 1 infection pathway was downloaded from the KEGG database (https://www.kegg.jp/kegg-bin/show_pathway?map05166). In K562 cells, HQ-upregulated DEGs were colored pink and HQ-downregulated DEGs were colored green.

## Data Availability

The datasets used and/or analyzed during the current study are available from the corresponding author on reasonable request.
