# Supplementary material for: Identification of potential pathways and microRNA-mRNA networks associated with benzene metabolite hydroquinone-induced hematotoxicity in human leukemia K562 cells
Source: BMC Pharmacol Toxicol. 2022 Apr 2;23:20. doi: 10.1186/s40360-022-00556-8 (PMC8976366; doi:10.1186/s40360-022-00556-8)

**Additional file 1**

**Supplementary Fig. 1**. HQ-regulated DEGs of K562 cells in the Wnt signaling pathway. The Wnt signaling pathway was downloaded from the KEGG database (<https://www.kegg.jp/kegg-bin/show_pathway?map04310>). In K562 cells, HQ-upregulated DEGs were colored pink and HQ-downregulated DEGs were colored green.


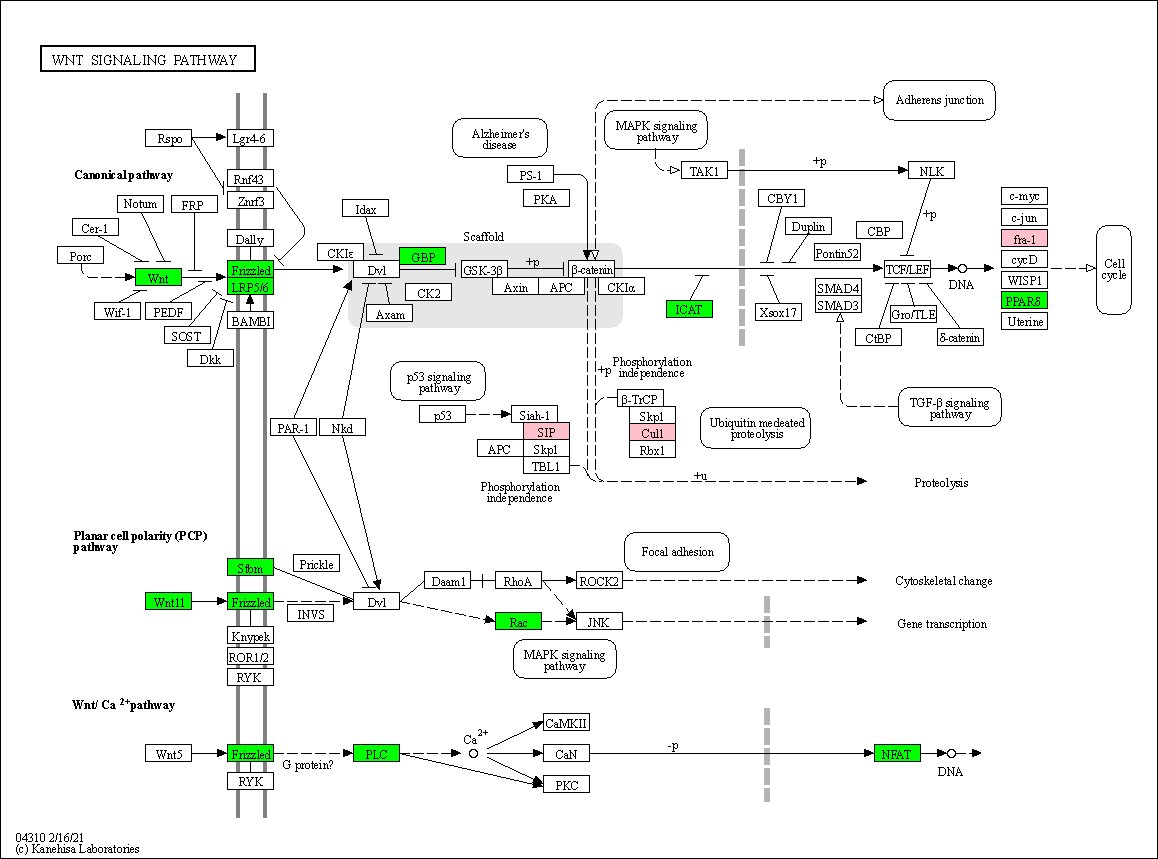


**Supplementary Fig. 2**. HQ-regulated DEGs of K562 cells in chronic myeloid leukemia pathway. The chronic myeloid leukemia pathway was downloaded from the KEGG database (<https://www.kegg.jp/kegg-bin/show_pathway?map05220>). In K562 cells, HQ-upregulated DEGs were colored pink and HQ-downregulated DEGs were colored green.


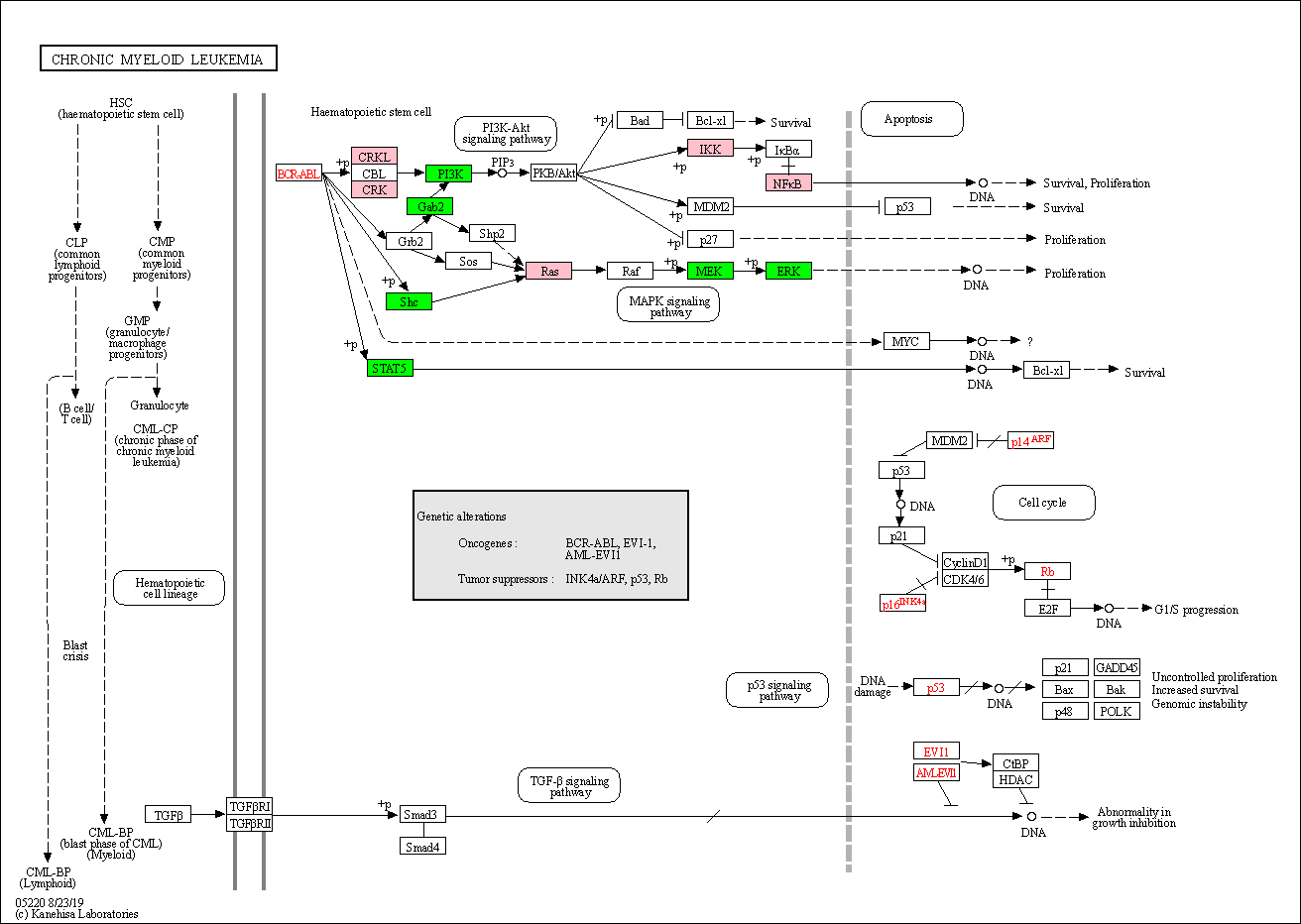


**Supplementary Fig. 3**. HQ-regulated DEGs of K562 cells in acute myeloid leukemia pathway. The acute myeloid leukemia pathway was downloaded from the KEGG database (<https://www.kegg.jp/kegg-bin/show_pathway?map05221>). In K562 cells, HQ-upregulated DEGs were colored pink and HQ-downregulated DEGs were colored green.


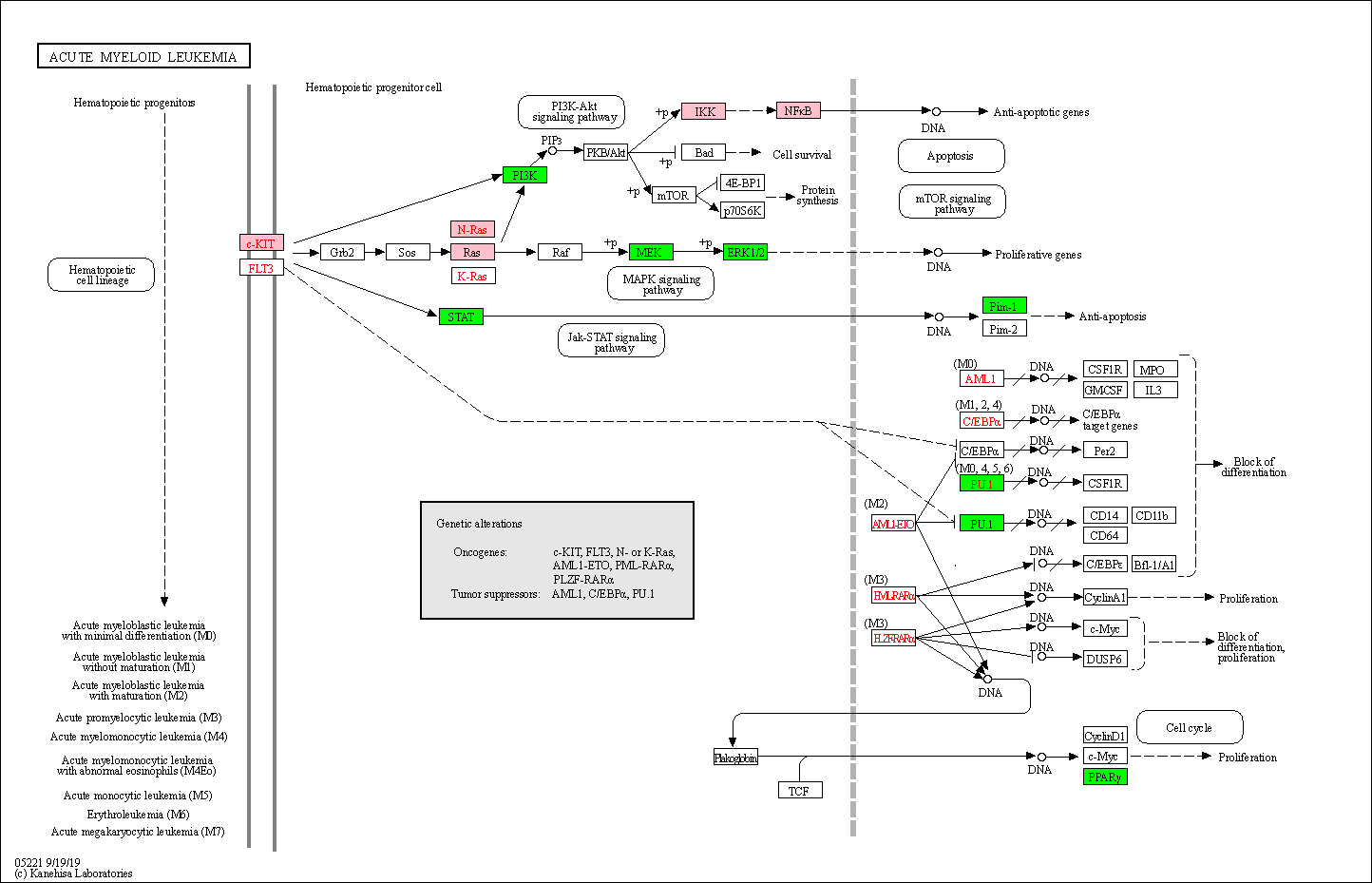


**Supplementary Fig. 4**. HQ-regulated DEGs of K562 cells in hematopoietic cell lineage pathway. The hematopoietic cell lineage pathway was downloaded from the KEGG database (<https://www.kegg.jp/kegg-bin/show_pathway?map04640>). In K562 cells, HQ-upregulated DEGs were colored pink and HQ-downregulated DEGs were colored green.


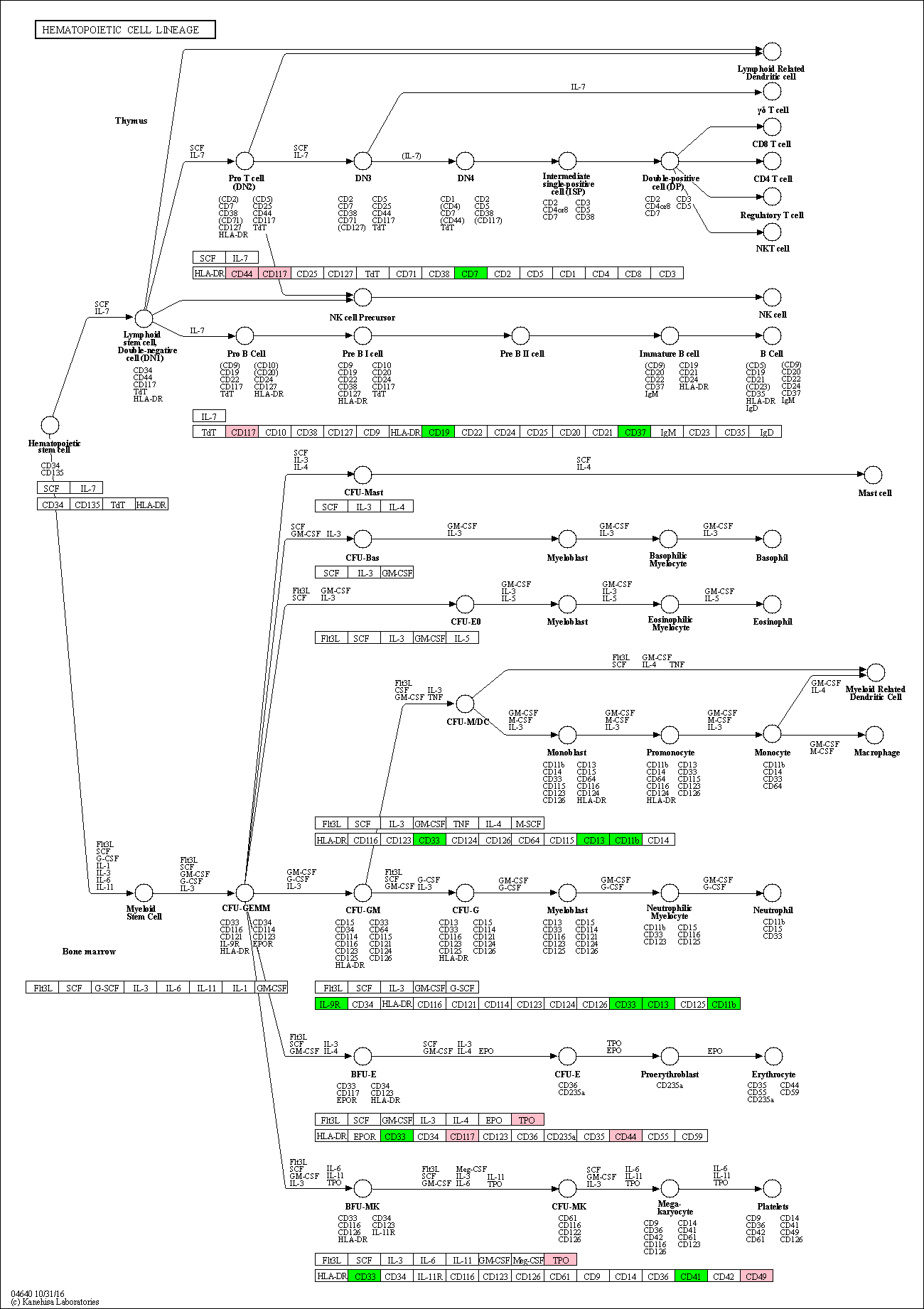


**Supplementary Fig. 5**. HQ-regulated DEGs of K562 cells in human T-cell leukemia virus 1 infection pathway. The human T-cell leukemia virus 1 infection pathway was downloaded from the KEGG database (<https://www.kegg.jp/kegg-bin/show_pathway?map05166>). In K562 cells, HQ-upregulated DEGs were colored pink and HQ-downregulated DEGs were colored green.


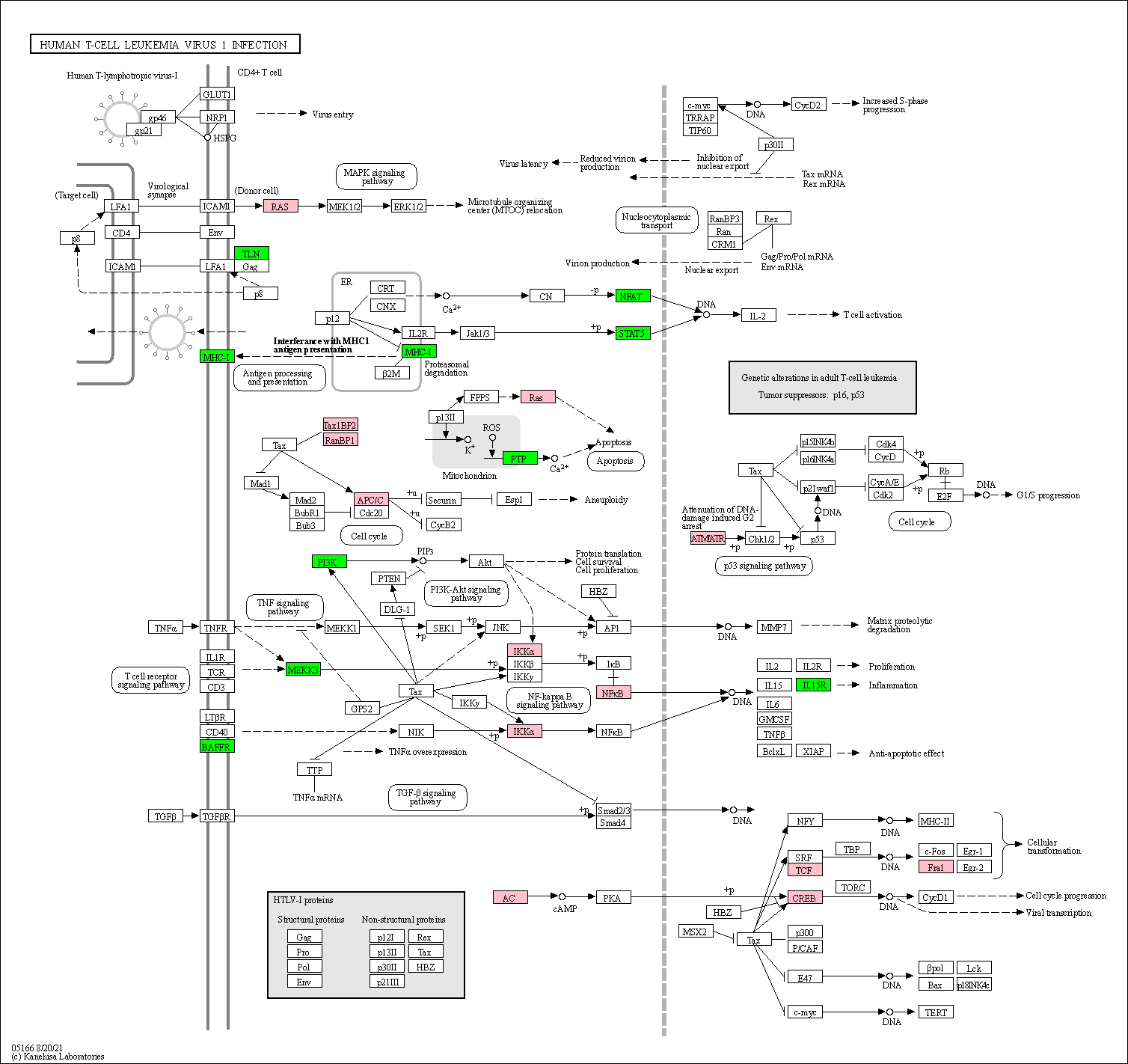

Supplement: Supplementary file 1 — Additional file 1: Supplementary Figure 1. HQ-regulated DEGs of K562 cells in the Wnt signaling pathway. The Wnt signaling pathway was downloaded from the KEGG database (https://www.kegg.jp/kegg-bin/show_pathway?map04310). In K562 cells, HQ-upregulated DEGs were colored pink and HQ-downregulated DEGs were colored green. Supplementary Figure 2. HQ-regulated DEGs of K562 cells in chronic myeloid leukemia pathway. The chronic myeloid leukemia pathway was downloaded from the KEGG database (https://www.kegg.jp/kegg-bin/show_pathway?map05220). In K562 cells, HQ-upregulated DEGs were colored pink and HQ-downregulated DEGs were colored green. Supplementary Figure 3. HQ-regulated DEGs of K562 cells in acute myeloid leukemia pathway. The acute myeloid leukemia pathway was downloaded from the KEGG database (https://www.kegg.jp/kegg-bin/show_pathway?map05221). In K562 cells, HQ-upregulated DEGs were colored pink and HQ-downregulated DEGs were colored green. Supplementary Figure 4. HQ-regulated DEGs of K562 cells in hematopoietic cell lineage pathway. The hematopoietic cell lineage pathway was downloaded from the KEGG database (https://www.kegg.jp/kegg-bin/show_pathway?map04640). In K562 cells, HQ-upregulated DEGs were colored pink and HQ-downregulated DEGs were colored green. Supplementary Figure 5. HQ-regulated DEGs of K562 cells in human T-cell leukemia virus 1 infection pathway. The human T-cell leukemia virus 1 infection pathway was downloaded from the KEGG database (https://www.kegg.jp/kegg-bin/show_pathway?map05166). In K562 cells, HQ-upregulated DEGs were colored pink and HQ-downregulated DEGs were colored green. [file 40360_2022_556_MOESM1_ESM.docx]
